# Editorial: Lipids and Inflammation in Health and Disease

**DOI:** 10.3389/fcvm.2022.864429

**Published:** 2022-03-15

**Authors:** Evgeny Bezsonov, Vasily Sukhorukov, Michael Bukrinsky, Alexander Orekhov

**Affiliations:** ^1^Laboratory of Cellular and Molecular Pathology of Cardiovascular System, A. P. Avtsyn Research Institute of Human Morphology, Moscow, Russia; ^2^Laboratory of Angiopathology, Institute of General Pathology and Pathophysiology, Moscow, Russia; ^3^Department of Biology and General Genetics, I. M. Sechenov First Moscow State Medical University (Sechenov University), Moscow, Russia; ^4^Department of Microbiology, Immunology and Tropical Medicine, School of Medicine and Health Sciences, The George Washington University, Washington, DC, United States; ^5^Skolkovo Innovative Center, Institute for Atherosclerosis Research, Moscow, Russia

**Keywords:** lipids, lipoproteins, inflammation, atherosclerosis, cholesterol

The focus of this Special Issue (SI) was the lipid-related activation of the innate and adaptive immune responses leading to the development of chronic inflammation, with particular attention paid to the pathogenesis of atherosclerosis. The key events in lipid-mediated regulation of immunity are the transport and metabolism of lipids of all possible classes, including lipid-containing particles (like lipoproteins), in different tissues and cells. Despite all progress during several decades of research on atherosclerosis, its pathogenesis is still far from being completely understood, limiting available therapeutic options. The current advances in therapy of this disease are related mainly to the use of statins, aimed at lowering cholesterol plasma levels, but still, this widely used approach does not provide desirable results in all clinical cases, reflecting more complex mechanisms behind atherosclerosis development.

The classic understanding of the initial stages of atherosclerosis involves modified low-density lipoproteins (LDL)-induced accumulation of lipids (cholesterol) in walls of large arteries. The atherogenesis-related LDL modifications include oxidation (it should be noted that there are different opinions on the role of this modification in atherogenesis) and often forgotten desialylation. A better understanding of the nature and mechanisms of chemical modifications of LDL particles leading to their atherogenicity can help to find new therapeutic options to treat atherosclerosis.

Another well established observation is an association between low levels of high-density lipoproteins (HDL) in blood plasma and increased risk of the development of cardiovascular diseases. This connection can be explained by the involvement of HDL in reverse cholesterol transport (removal of this lipid from the walls of arteries). Based on these clinical findings, HDL is considered to be an important target for anti-atherosclerotic therapy.

In addition to lipoprotein disbalance, chronic inflammation is another important pathogenic factor in atherosclerosis. The specifics of atherosclerosis-associated inflammation, and especially the connection between inflammation and accumulation of lipids are the current hot topics of research. Recent findings that certain mutations of mitochondrial DNA (mtDNA) are associated with atherosclerotic lesions suggest that these mutations (induced or spontaneous) may serve as a trigger of inflammation in arterial walls: the defective mitophagy may activate innate immune responses leading to secretion of inflammatory cytokines and non-resolving inflammation. This increases the accumulation of lipids in the artery walls (initially induced by modified LDL) and, as a result, promotes the formation of advanced atherosclerotic plaque. This hypothesis is shown in Figure 1.

**Figure 1:**
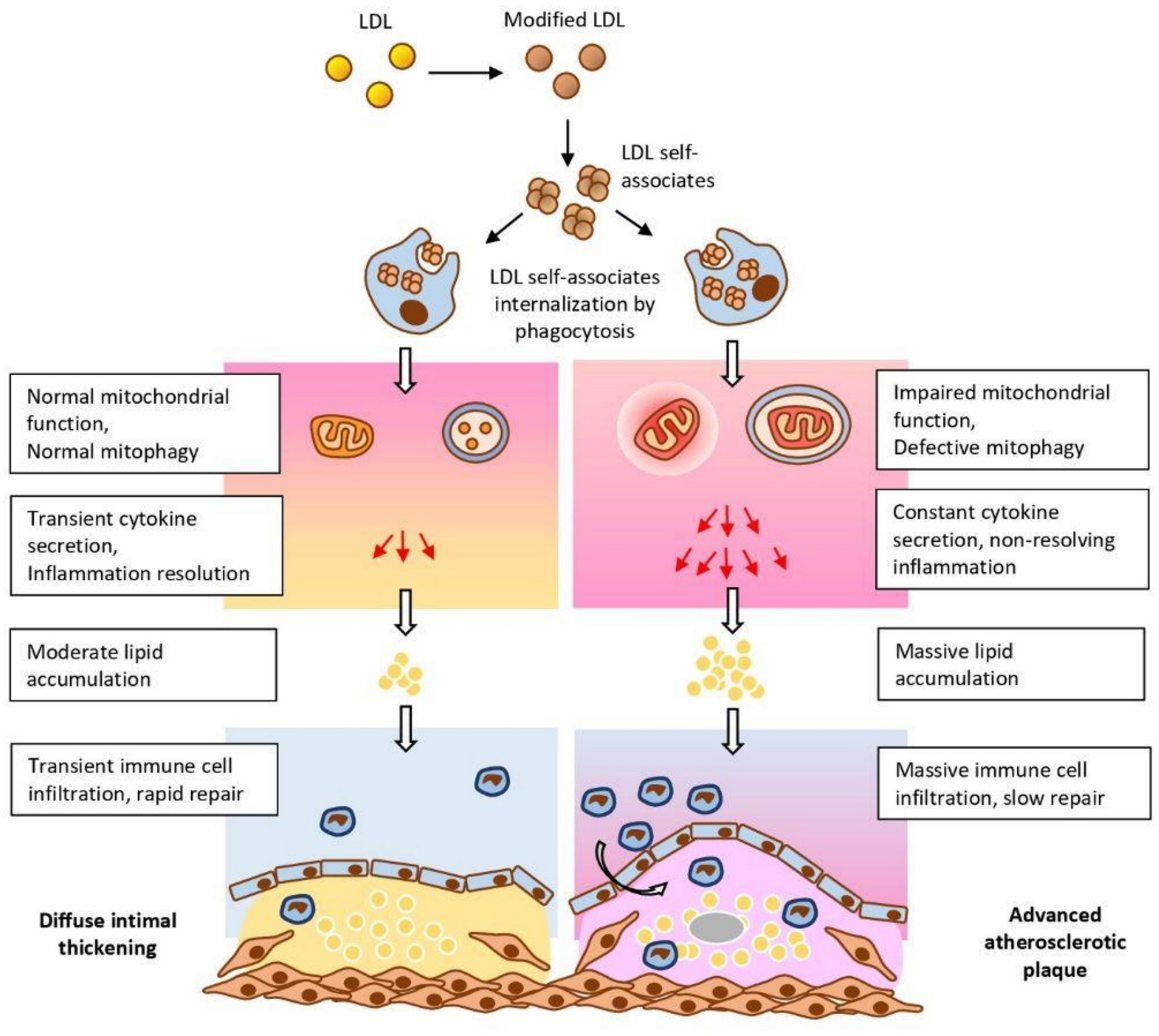
The influence of mtDNA mutations on the development of atherosclerosis. Mutations may enhance modified LDL-induced lipid accumulation in arterial walls by stimulation of innate immune responses, and, as a result, the establishment of non-resolving inflammation (1) [the figure was borrowed from the publication (1) distributed under Creative Commons Attribution License].

The current SI has several research papers and reviews devoted to the above-mentioned hypothesis, as well as to other topics related to atherogenesis; below, we will briefly describe each contribution underscoring the key findings.

Using the model of ob/ob mice, Saito et al. demonstrated that α-Galactosylceramide-induced activation of iNKT cells was able to reduce the development of aortic aneurism induced by angiotensin II. This study suggests that activation of iNKT cells may be a novel therapeutic target against the development of abdominal aortic aneurism.

Arida et al. discovered that proprotein convertase subtilisin/kexin type 9 (PCSK9) and LDL-Receptor (LDLR) levels correlated with the presence of plaques in patients with rheumatoid arthritis (RA), suggesting a significant involvement of PCSK9/LDLR system in RA-associated atherosclerosis development.

Bestavashvili et al. have tried to assess the influence of interval hypoxic-hyperoxic training (IHHT) on the lipid profile and inflammation in patients with metabolic syndrome. They conclude lipid profile and anti-inflammatory status of patients are impacted after IHHT procedure.

The pro-atherogenic role of Th17 cells was confirmed by Wang et al. using apoE^−/−^ mouse model of atherosclerosis and human blood samples from hyperlipidemic patients. They conclude that Th17 cells promote inflammation, and IL-17 is a potential target for anti-atherosclerotic drugs.

In the report by Ganjooei et al. it was found that lipid profiles based on total cholesterol, LDL-C, HDL-C, VLDL, and triglycerides in morbidly obese patients were not associated with non-alcoholic steatohepatitis (NASH) and liver fibrosis. These results suggest that other factors may play the primary role in these morbidities, especially at the late stages investigated in this report.

Wang et al. report an observation that monocyte-to-lymphocyte ratio (MLR) is a high-risk factor related to arterial stiffness and could serve as a potential marker of this pathology.

Using the hypoxia rat model of severe pulmonary arterial hypertension (PAH), Spyropoulos et al. demonstrated that acetazolamide added to drinking water inhibited inflammation and prevented the development of PAH, right ventricular hypertrophy (RVH), and fibrosis.

Chidambaram et al. reported that high levels of cholesterol in serum of patients with tuberculosis (TB) were associated with decreased mortality and systemic inflammation with no correlation with body mass index (BMI). These counterintuitive results illustrate the fact that the effect of cholesterol on inflammation is multi-faceted and varies between different conditions and diseases.

Donis et al. demonstrated in a rabbit model that intake of palmitic acid (PA) associates with calcification of the aortic valve. This study raises the awareness about potential negative effects of PA dietary consumption.

Using molecular dynamics simulation, Ayee and Levitan found that even a slight increase in sterol (cholesterol and 7-ketocholesterol) levels in the membrane may lead to significant changes in the membrane structure, supposedly affecting biochemical, biophysical, and functional properties of the membrane.

In the model of normotensive and hypertensive rats, Schreckenberg et al. discovered that spironolactone-induced reduction of the number of neutrophils was blunted upon its combination with high physical activity in hypertensive rats. This study demonstrates that, although high physical activity has beneficial effects in normotensive subjects, care has to be taken on interactions between pharmacological approaches and high physical activity in hypertensives.

Hartley et al. reported that they found no effect of a percutaneous coronary intervention (PCI) on exercise-induced reduction of anti-malondialdehyde-LDL antibodies in the case of patients with stable coronary artery disease. They conclude that while exercise results in an acute reduction in anti-oxLDL antibodies in patients with severe single vessel coronary disease, PCI did not ameliorate this effect.

Wang et al. reported that chronic intermittent hypoxia led to the activation of brown adipose tissue (BAT) and increased atherosclerotic plaque size with the increase of CD68, α-SMA, and collagen in apoE^−/−^ mouse model of atherosclerosis. This study reveals the role of BAT in the pathogenesis of atherosclerosis.

Using a mice model deficient in ApoE gene, in which hypertension was induced by angiotensin II, Zhang et al. reported that a 2-week administration of RIPK1 inhibitor led to a reduction of the size of atherosclerotic lesions, while continuation of this treatment caused the increased formation of the lesions at 4 weeks. The results suggest that the duration of potential therapeutic usage of RIPK1 inhibitors should be chosen carefully.

Meng et al. analyzed proteins specific for different types of human coronary heart diseases associated with atherosclerosis. They identified six proteins related to cholesterol metabolism: Alb, Shbg, Apoc2, Apoc3, Apoc4, and Saa4, that can be used as markers of specific variations of coronary heart disease.

A report by Ng et al. describes results of high-intensity statin therapy in Asian patients subjected to percutaneous coronary intervention (PCI). They reported that statin treatment was associated with lower adjusted risk of major adverse cardiac event during 5 years after PCI, regardless of achieving the guideline's recommended levels of LDL-C, in comparison with patients who achieved recommended LDL-C target values without the application of high intensity statin therapy. Results illustrate the therapeutic value of high intensity statin treatment.

The study by Dong et al. of 4,128 subjects reported counterintuitive results that both increased and decreased levels of total cholesterol, decreased levels of ApoB, and increased levels of ApoAI can be associated with increased risk of cardiovascular diseases, whereas decreased levels of triglycerides can be associated with the increased mortality. These results suggest that maintaining optimal lipid levels is necessary for treatment strategies.

A review article by Zeng et al. summarized and reviewed the risk factors of Kawasaki disease with the focus on epidemiology, pathology (including connection with atherosclerosis), and long-term management of this disease.

The article by Shemiakova et al. reviews the role of mutations of mitochondrial DNA in the development of atherosclerosis. Possible ways to provide anti-atherosclerotic therapy through targeting mitochondrial pathologies are also reviewed and discussed.

Current views on the role of defects in the lymphatic system in hypercholesterolemia and atherosclerosis are reviewed in the article by Miyazaki and Miyazaki.

The influence of age and gender on the development and pathogenesis of atherosclerosis is discussed in the article by Vakhtangadze et al.

The review by Ma et al. provides a systematic analysis of quantitative nuclear magnetic resonance biomarkers of atherosclerosis and cardiovascular diseases with the focus on lipoproteins.

The mechanisms (on molecular and cellular levels) of atherosclerosis development in relation to lipid accumulation and inflammation are reviewed and discussed in the article by Malekmohammad et al. with a section devoted to the role of medicinal plants in the treatment of atherosclerosis.

The article by Li et al. presents new views on the mechanisms of function of the transcription factor-7-like-2 (TCF7L2) with the focus on its anti-atherosclerotic activities, as well as its potential as a new drug target for therapy of atherosclerosis.

A review by Zhang et al. is devoted to the mechanisms of action and potential for application of sinomenine in the treatment of cardio- and cerebrovascular diseases (including atherosclerosis).

We hope that the next SI will continue traditions of high-quality research and reviews established by the current one.

## Author Contributions

EB and AO: writing and editing of the manuscript. MB and VS: editing of the manuscript. All authors contributed to the article and approved the submitted version.

## Funding

Research was supported by the Russian Science Foundation, Grant Number 20-15-00264.

## Conflict of Interest

The authors declare that the research was conducted in the absence of any commercial or financial relationships that could be construed as a potential conflict of interest.

## Publisher's Note

All claims expressed in this article are solely those of the authors and do not necessarily represent those of their affiliated organizations, or those of the publisher, the editors and the reviewers. Any product that may be evaluated in this article, or claim that may be made by its manufacturer, is not guaranteed or endorsed by the publisher.
